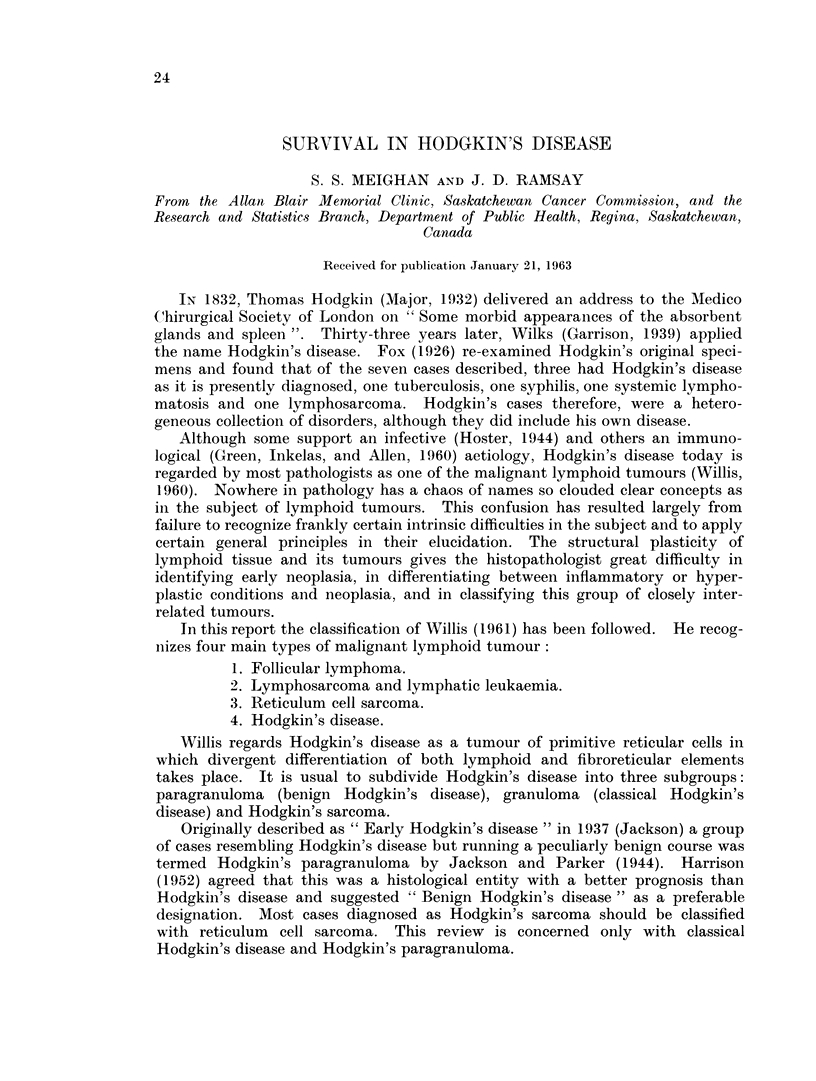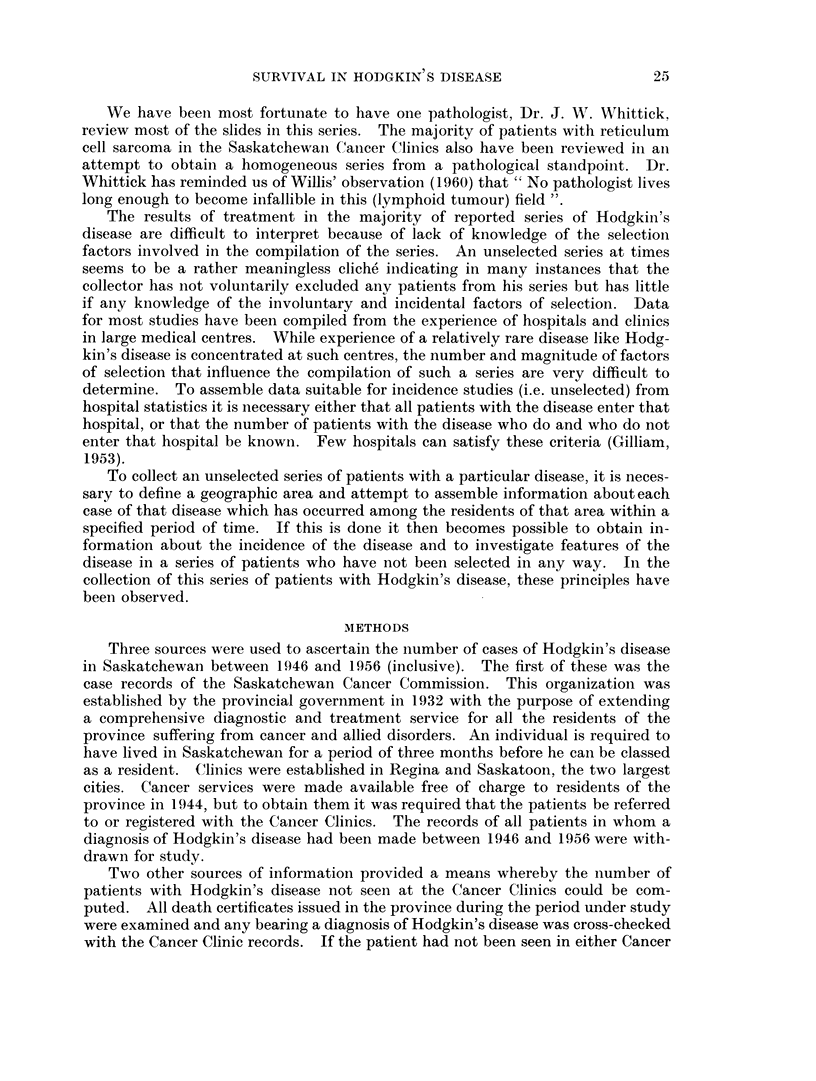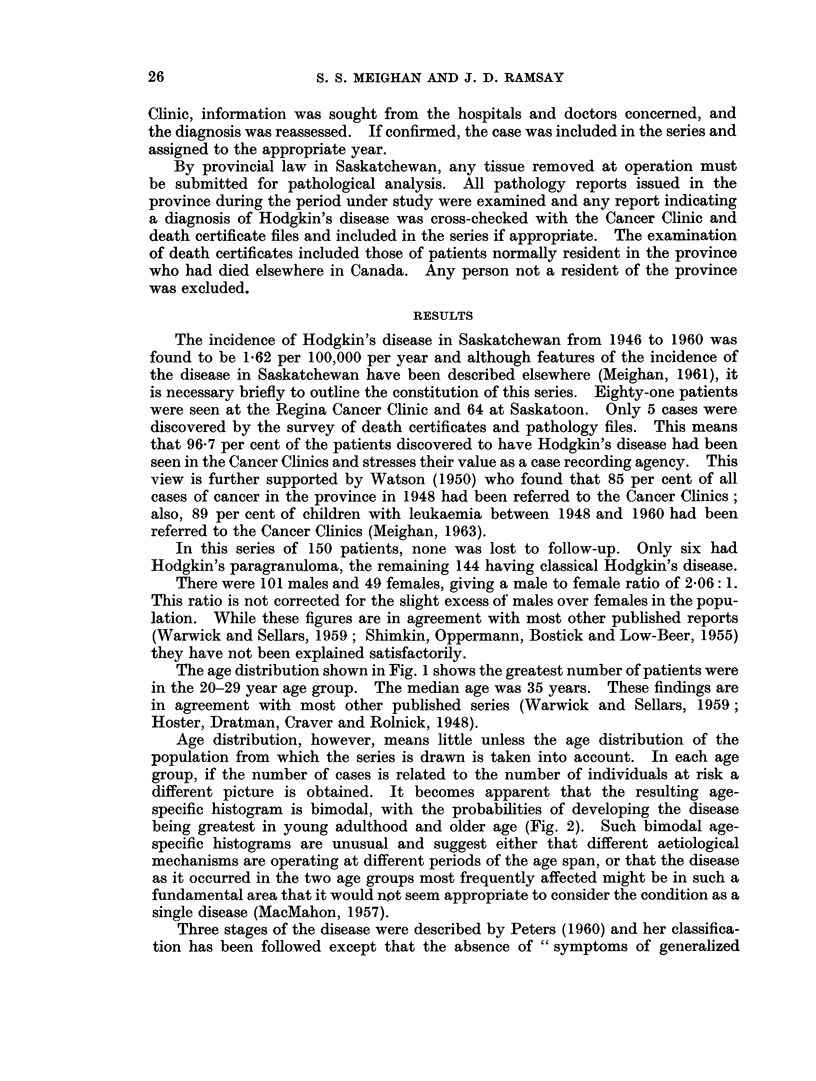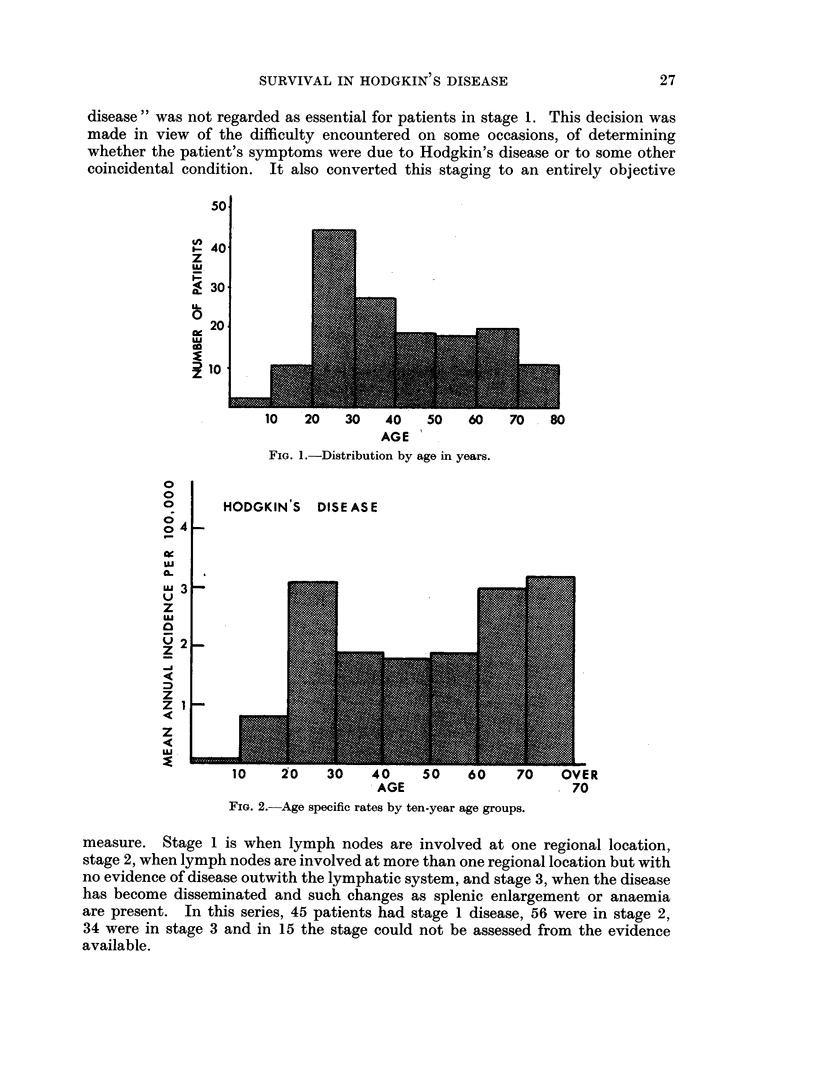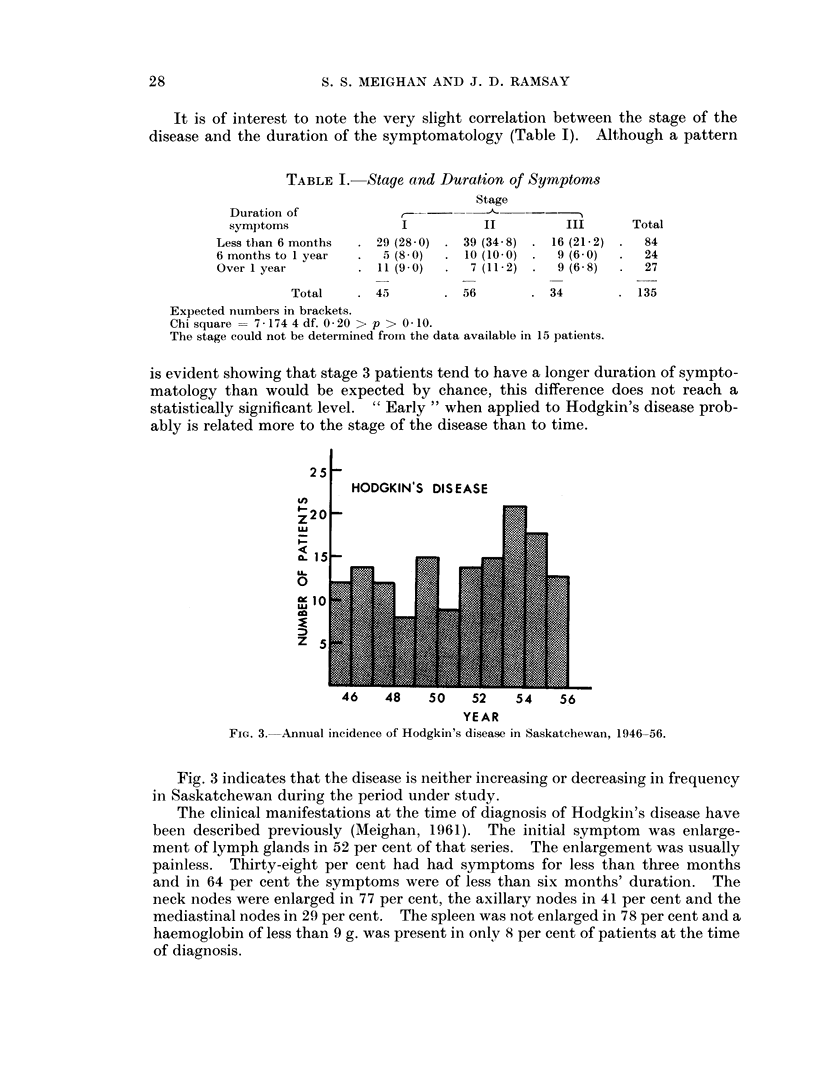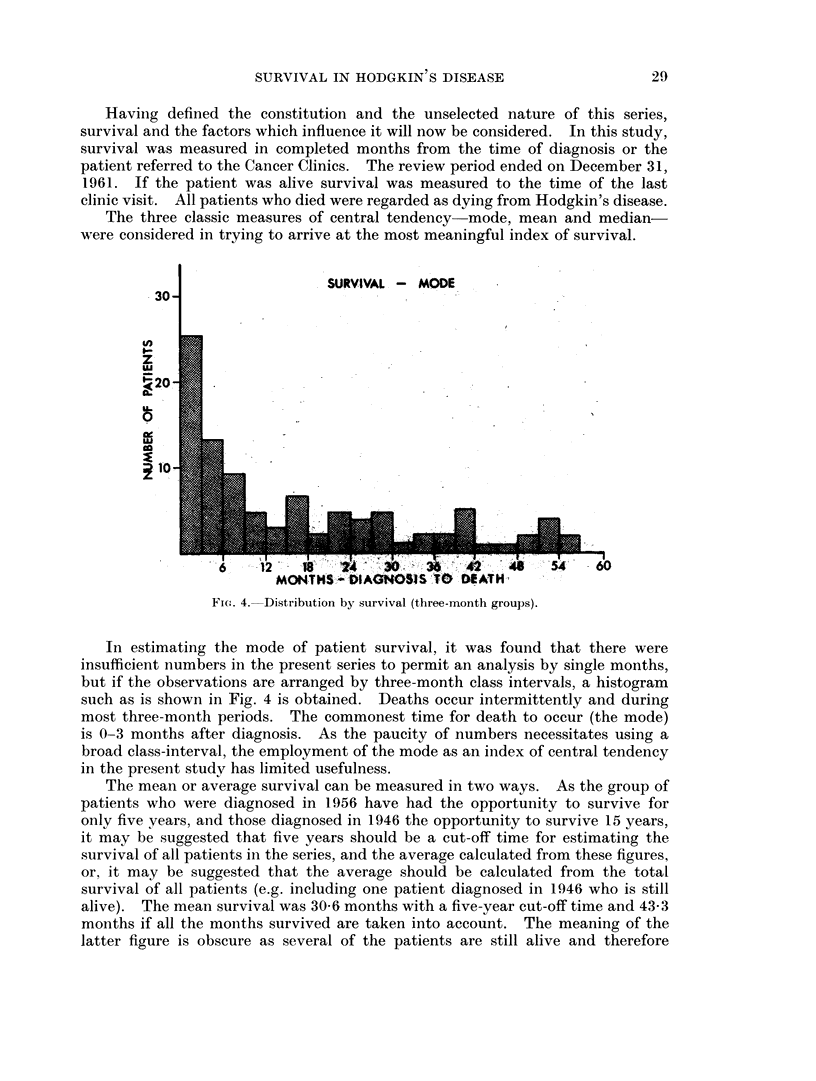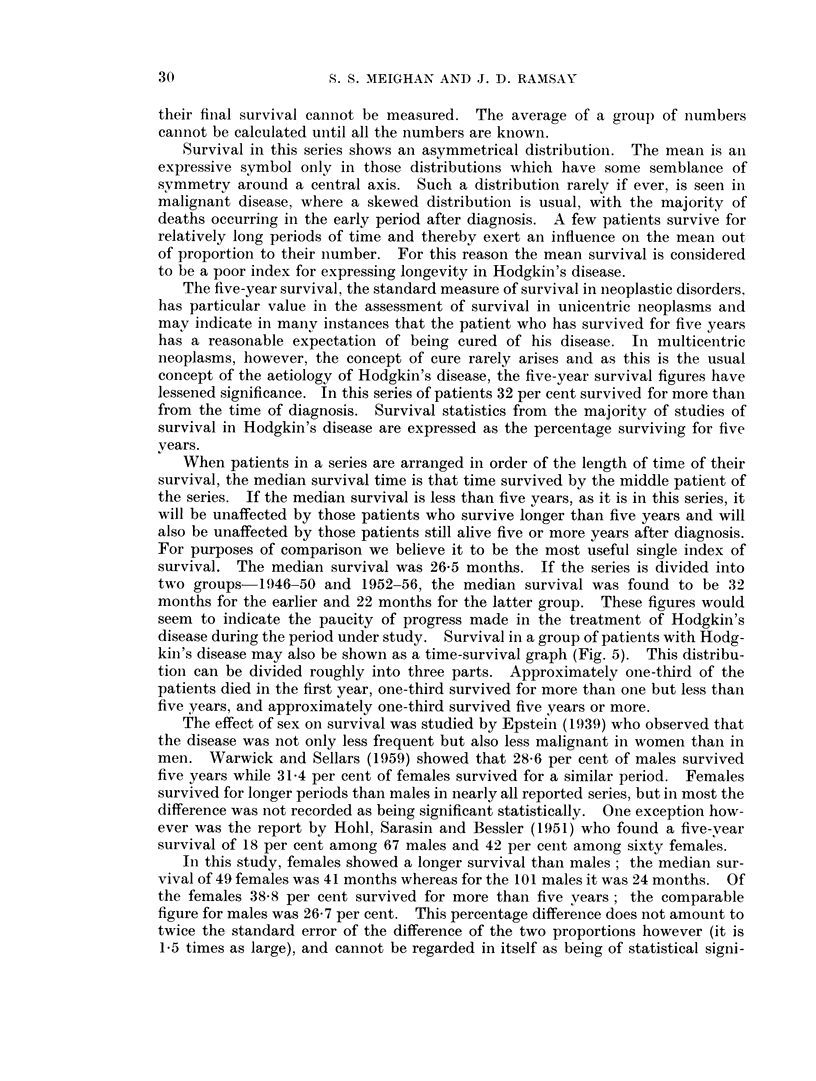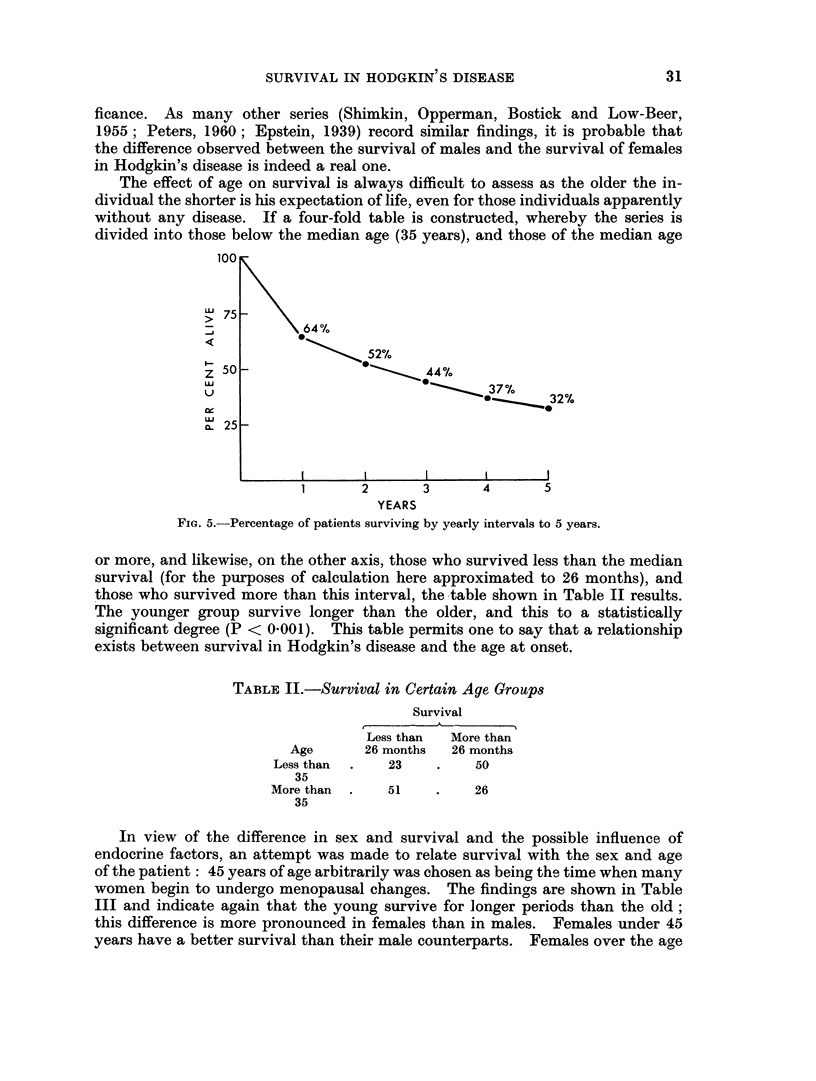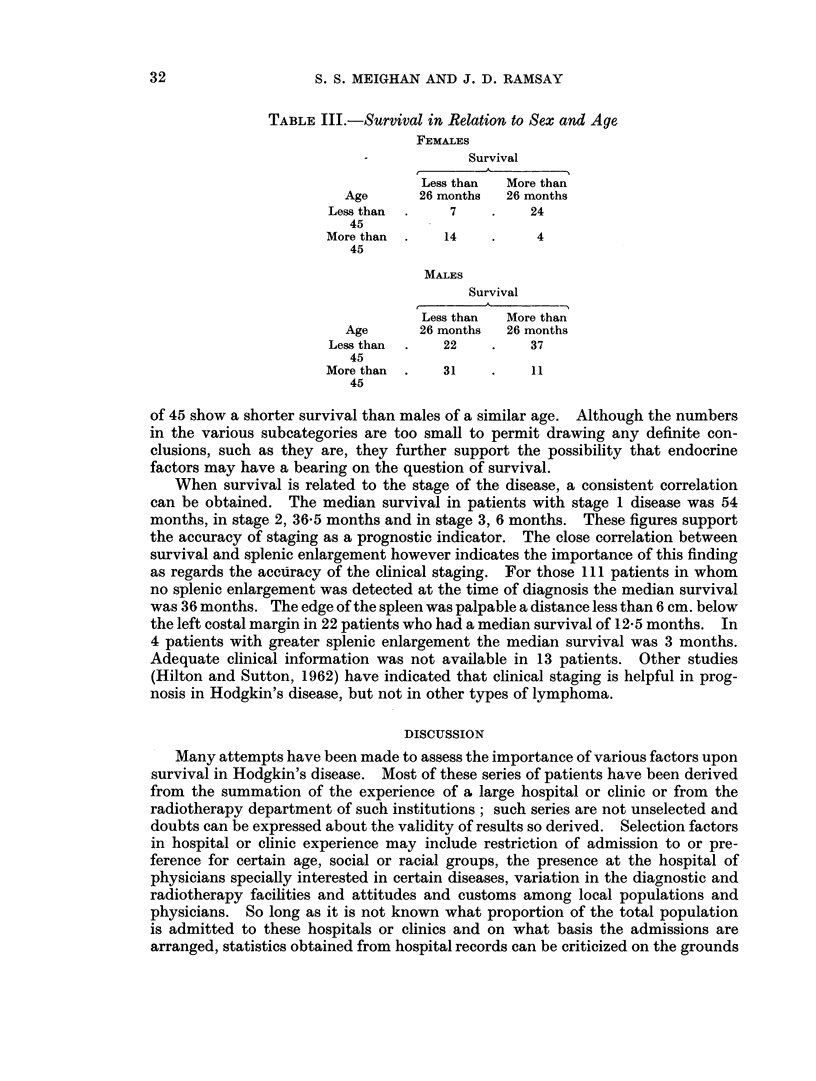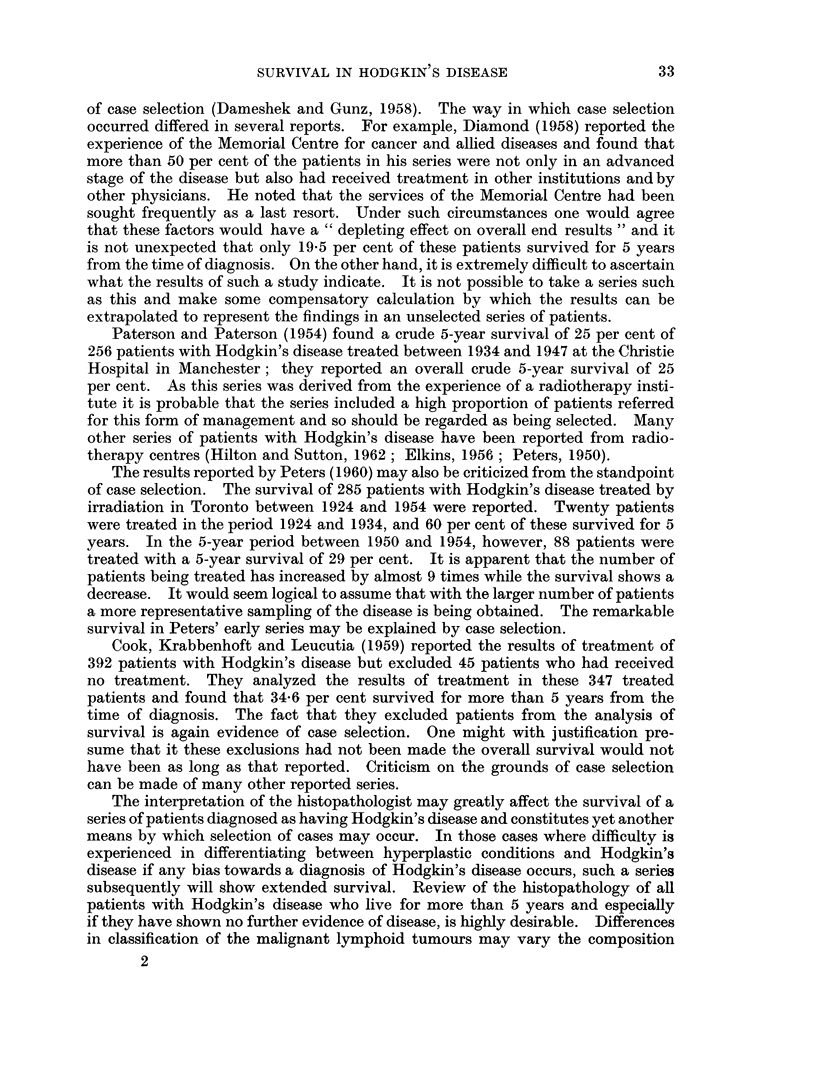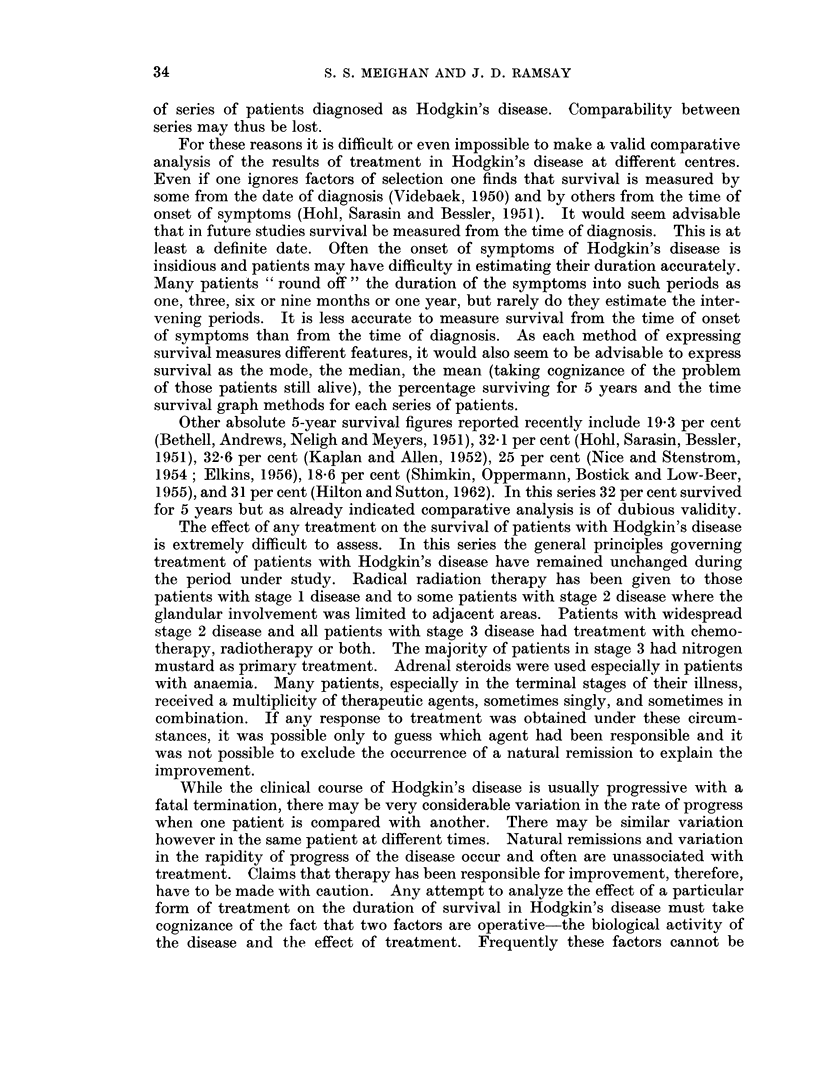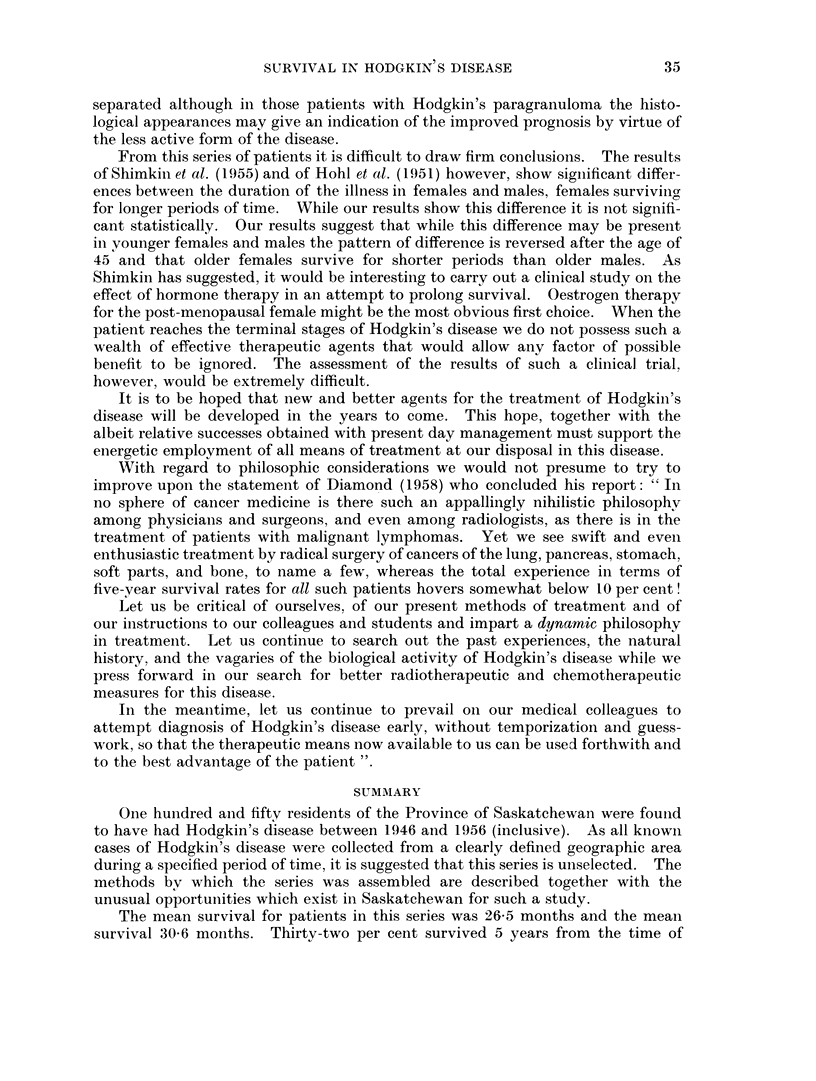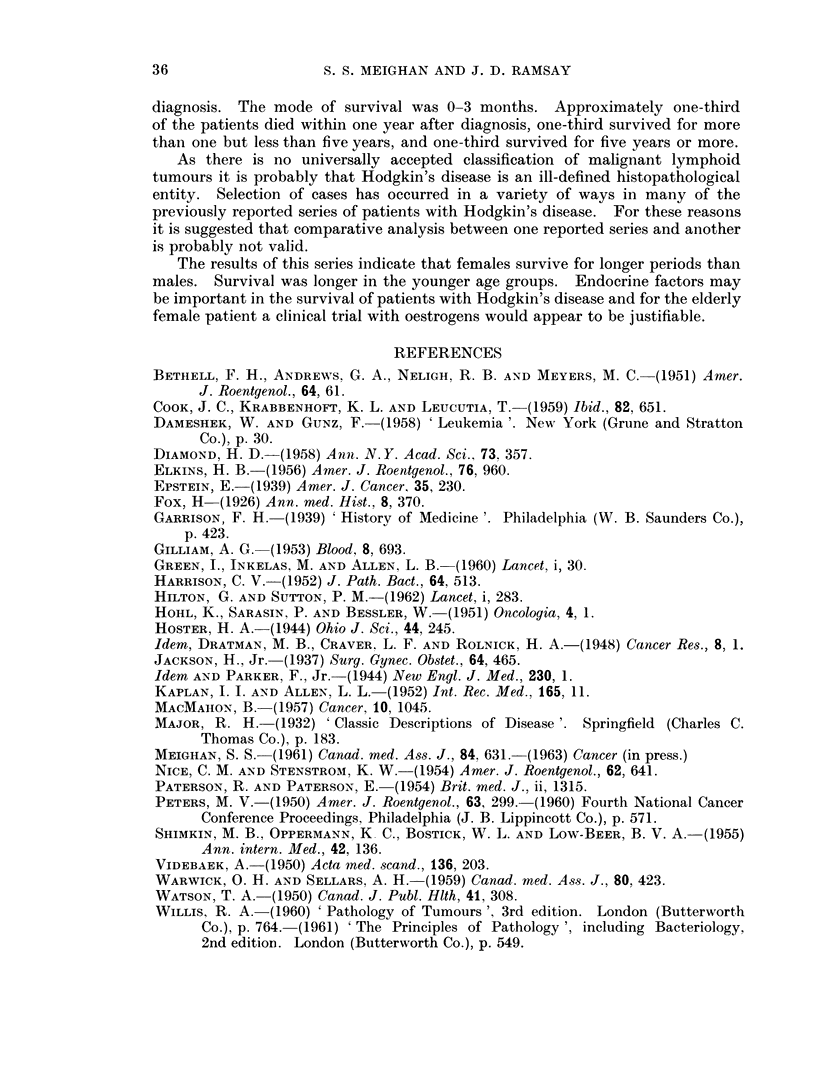# Survival in Hodgkin's Disease

**DOI:** 10.1038/bjc.1963.3

**Published:** 1963-03

**Authors:** S. S. Meighan, J. D. Ramsay


### SURVIVAL IN HODGKIN'S DISEASE

S. S. MEIGHAN AND J. D. RAMSAY

*From the Allan Blair Memorial Clinic, Saskatchewan Cancer Commission, and the Research and Statistics Branch, Department of Public Health, Regina, Saskatchewan, Canada*

Received for publication January 21, 1963

IN 1832, Thomas Hodgkin (Major, 1932) delivered an address to the Medico Chirurgical Society of London on " Some morbid appearances of the absorbent glands and spleen ". Thirty-three years later, Wilks (Garrison, 1939) applied the name Hodgkin's disease. Fox (1926) re-examined Hodgkin's original specimens and found that of the seven cases described, three had Hodgkin's disease as it is presently diagnosed, one tuberculosis, one syphilis, one systemic lymphomatosis and one lymphosarcoma. Hodgkin's cases therefore, were a heterogeneous collection of disorders, although they did include his own disease.

Although some support an infective (Hoster, 1944) and others an immunological (Green, Inkelas, and Allen, 1960) aetiology, Hodgkin's disease today is regarded by most pathologists as one of the malignant lymphoid tumours (Willis, 1960). Nowhere in pathology has a chaos of names so clouded clear concepts as in the subject of lymphoid tumours. This confusion has resulted largely from failure to recognize frankly certain intrinsic difficulties in the subject and to apply certain general principles in their elucidation. The structural plasticity of lymphoid tissue and its tumours gives the histopathologist great difficulty in identifying early neoplasia, in differentiating between inflammatory or hyperplastic conditions and neoplasia, and in classifying this group of closely interrelated tumours.

In this report the classification of Willis (1961) has been followed. He recognizes four main types of malignant lymphoid tumour :

1. Follicular lymphoma.
2. Lymphosarcoma and lymphatic leukaemia.
3. Reticulum cell sarcoma.
4. Hodgkin's disease.

Willis regards Hodgkin's disease as a tumour of primitive reticular cells in which divergent differentiation of both lymphoid and fibroreticular elements takes place. It is usual to subdivide Hodgkin's disease into three subgroups: paragranuloma (benign Hodgkin's disease), granuloma (classical Hodgkin's disease) and Hodgkin's sarcoma.

Originally described as " Early Hodgkin's disease " in 1937 (Jackson) a group of cases resembling Hodgkin's disease but running a peculiarly benign course was termed Hodgkin's paragranuloma by Jackson and Parker (1944). Harrison (1952) agreed that this was a histological entity with a better prognosis than Hodgkin's disease and suggested " Benign Hodgkin's disease " as a preferable designation. Most cases diagnosed as Hodgkin's sarcoma should be classified with reticulum cell sarcoma. This review is concerned only with classical Hodgkin's disease and Hodgkin's paragranuloma.

We have been most fortunate to have one pathologist, Dr. J. W. Whittick, review most of the slides in this series. The majority of patients with reticulum cell sarcoma in the Saskatchewan Cancer Clinics also have been reviewed in an attempt to obtain a homogeneous series from a pathological standpoint. Dr. Whittick has reminded us of Willis' observation (1960) that " No pathologist lives long enough to become infallible in this (lymphoid tumour) field ".

The results of treatment in the majority of reported series of Hodgkin's disease are difficult to interpret because of lack of knowledge of the selection factors involved in the compilation of the series. An unselected series at times seems to be a rather meaningless cliché indicating in many instances that the collector has not voluntarily excluded any patients from his series but has little if any knowledge of the involuntary and incidental factors of selection. Data for most studies have been compiled from the experience of hospitals and clinics in large medical centres. While experience of a relatively rare disease like Hodgkin's disease is concentrated at such centres, the number and magnitude of factors of selection that influence the compilation of such a series are very difficult to determine. To assemble data suitable for incidence studies (i.e. unselected) from hospital statistics it is necessary either that all patients with the disease enter that hospital, or that the number of patients with the disease who do and who do not enter that hospital be known. Few hospitals can satisfy these criteria (Gilliam, 1953).

To collect an unselected series of patients with a particular disease, it is necessary to define a geographic area and attempt to assemble information about each case of that disease which has occurred among the residents of that area within a specified period of time. If this is done it then becomes possible to obtain information about the incidence of the disease and to investigate features of the disease in a series of patients who have not been selected in any way. In the collection of this series of patients with Hodgkin's disease, these principles have been observed.

### METHODS

Three sources were used to ascertain the number of cases of Hodgkin's disease in Saskatchewan between 1946 and 1956 (inclusive). The first of these was the case records of the Saskatchewan Cancer Commission. This organization was established by the provincial government in 1932 with the purpose of extending a comprehensive diagnostic and treatment service for all the residents of the province suffering from cancer and allied disorders. An individual is required to have lived in Saskatchewan for a period of three months before he can be classed as a resident. Clinics were established in Regina and Saskatoon, the two largest cities. Cancer services were made available free of charge to residents of the province in 1944, but to obtain them it was required that the patients be referred to or registered with the Cancer Clinics. The records of all patients in whom a diagnosis of Hodgkin's disease had been made between 1946 and 1956 were withdrawn for study.

Two other sources of information provided a means whereby the number of patients with Hodgkin's disease not seen at the Cancer Clinics could be computed. All death certificates issued in the province during the period under study were examined and any bearing a diagnosis of Hodgkin's disease was cross-checked with the Cancer Clinic records. If the patient had not been seen in either Cancer

Clinic, information was sought from the hospitals and doctors concerned, and the diagnosis was reassessed. If confirmed, the case was included in the series and assigned to the appropriate year.

By provincial law in Saskatchewan, any tissue removed at operation must be submitted for pathological analysis. All pathology reports issued in the province during the period under study were examined and any report indicating a diagnosis of Hodgkin's disease was cross-checked with the Cancer Clinic and death certificate files and included in the series if appropriate. The examination of death certificates included those of patients normally resident in the province who had died elsewhere in Canada. Any person not a resident of the province was excluded.

### RESULTS

The incidence of Hodgkin's disease in Saskatchewan from 1946 to 1960 was found to be 1·62 per 100,000 per year and although features of the incidence of the disease in Saskatchewan have been described elsewhere (Meighan, 1961), it is necessary briefly to outline the constitution of this series. Eighty-one patients were seen at the Regina Cancer Clinic and 64 at Saskatoon. Only 5 cases were discovered by the survey of death certificates and pathology files. This means that 96·7 per cent of the patients discovered to have Hodgkin's disease had been seen in the Cancer Clinics and stresses their value as a case recording agency. This view is further supported by Watson (1950) who found that 85 per cent of all cases of cancer in the province in 1948 had been referred to the Cancer Clinics; also, 89 per cent of children with leukaemia between 1948 and 1960 had been referred to the Cancer Clinics (Meighan, 1963).

In this series of 150 patients, none was lost to follow-up. Only six had Hodgkin's paragranuloma, the remaining 144 having classical Hodgkin's disease.

There were 101 males and 49 females, giving a male to female ratio of 2·06 : 1. This ratio is not corrected for the slight excess of males over females in the population. While these figures are in agreement with most other published reports (Warwick and Sellars, 1959 ; Shimkin, Oppermann, Bostick and Low-Beer, 1955) they have not been explained satisfactorily.

The age distribution shown in Fig. 1 shows the greatest number of patients were in the 20–29 year age group. The median age was 35 years. These findings are in agreement with most other published series (Warwick and Sellars, 1959 ; Hoster, Dratman, Craver and Rolnick, 1948).

Age distribution, however, means little unless the age distribution of the population from which the series is drawn is taken into account. In each age group, if the number of cases is related to the number of individuals at risk a different picture is obtained. It becomes apparent that the resulting age-specific histogram is bimodal, with the probabilities of developing the disease being greatest in young adulthood and older age (Fig. 2). Such bimodal age-specific histograms are unusual and suggest either that different aetiological mechanisms are operating at different periods of the age span, or that the disease as it occurred in the two age groups most frequently affected might be in such a fundamental area that it would not seem appropriate to consider the condition as a single disease (MacMahon, 1957).

Three stages of the disease were described by Peters (1960) and her classification has been followed except that the absence of " symptoms of generalized disease" was not regarded as essential for patients in stage 1. This decision was made in view of the difficulty encountered on some occasions, of determining whether the patient's symptoms were due to Hodgkin's disease or to some other coincidental condition. It also converted this staging to an entirely objective

FIG. 1.—Distribution by age in years.

FIG. 2.—Age specific rates by ten-year age groups.

measure. Stage 1 is when lymph nodes are involved at one regional location, stage 2, when lymph nodes are involved at more than one regional location but with no evidence of disease outwith the lymphatic system, and stage 3, when the disease has become disseminated and such changes as splenic enlargement or anaemia are present. In this series, 45 patients had stage 1 disease, 56 were in stage 2, 34 were in stage 3 and in 15 the stage could not be assessed from the evidence available.

It is of interest to note the very slight correlation between the stage of the disease and the duration of the symptomatology (Table I). Although a pattern

TABLE I.—*Stage and Duration of Symptoms*

| Duration of symptoms | Stage I | Stage II | Stage III | Total |
|---|---|---|---|---|
| Less than 6 months | 29 (28·0) | 39 (34·8) | 16 (21·2) | 84 |
| 6 months to 1 year | 5 (8·0) | 10 (10·0) | 9 (6·0) | 24 |
| Over 1 year | 11 (9·0) | 7 (11·2) | 9 (6·8) | 27 |
| Total | 45 | 56 | 34 | 135 |

Expected numbers in brackets.
Chi square = 7·174 4 df. $0\cdot20 > p > 0\cdot10$.
The stage could not be determined from the data available in 15 patients.

is evident showing that stage 3 patients tend to have a longer duration of symptomatology than would be expected by chance, this difference does not reach a statistically significant level. " Early " when applied to Hodgkin's disease probably is related more to the stage of the disease than to time.

FIG. 3.—Annual incidence of Hodgkin's disease in Saskatchewan, 1946–56.

Fig. 3 indicates that the disease is neither increasing or decreasing in frequency in Saskatchewan during the period under study.

The clinical manifestations at the time of diagnosis of Hodgkin's disease have been described previously (Meighan, 1961). The initial symptom was enlargement of lymph glands in 52 per cent of that series. The enlargement was usually painless. Thirty-eight per cent had had symptoms for less than three months and in 64 per cent the symptoms were of less than six months' duration. The neck nodes were enlarged in 77 per cent, the axillary nodes in 41 per cent and the mediastinal nodes in 29 per cent. The spleen was not enlarged in 78 per cent and a haemoglobin of less than 9 g. was present in only 8 per cent of patients at the time of diagnosis.

Having defined the constitution and the unselected nature of this series, survival and the factors which influence it will now be considered. In this study, survival was measured in completed months from the time of diagnosis or the patient referred to the Cancer Clinics. The review period ended on December 31, 1961. If the patient was alive survival was measured to the time of the last clinic visit. All patients who died were regarded as dying from Hodgkin's disease.

The three classic measures of central tendency—mode, mean and median—were considered in trying to arrive at the most meaningful index of survival.

Fig. 4.—Distribution by survival (three-month groups).

In estimating the mode of patient survival, it was found that there were insufficient numbers in the present series to permit an analysis by single months, but if the observations are arranged by three-month class intervals, a histogram such as is shown in Fig. 4 is obtained. Deaths occur intermittently and during most three-month periods. The commonest time for death to occur (the mode) is 0–3 months after diagnosis. As the paucity of numbers necessitates using a broad class-interval, the employment of the mode as an index of central tendency in the present study has limited usefulness.

The mean or average survival can be measured in two ways. As the group of patients who were diagnosed in 1956 have had the opportunity to survive for only five years, and those diagnosed in 1946 the opportunity to survive 15 years, it may be suggested that five years should be a cut-off time for estimating the survival of all patients in the series, and the average calculated from these figures, or, it may be suggested that the average should be calculated from the total survival of all patients (e.g. including one patient diagnosed in 1946 who is still alive). The mean survival was 30·6 months with a five-year cut-off time and 43·3 months if all the months survived are taken into account. The meaning of the latter figure is obscure as several of the patients are still alive and therefore their final survival cannot be measured. The average of a group of numbers cannot be calculated until all the numbers are known.

Survival in this series shows an asymmetrical distribution. The mean is an expressive symbol only in those distributions which have some semblance of symmetry around a central axis. Such a distribution rarely if ever, is seen in malignant disease, where a skewed distribution is usual, with the majority of deaths occurring in the early period after diagnosis. A few patients survive for relatively long periods of time and thereby exert an influence on the mean out of proportion to their number. For this reason the mean survival is considered to be a poor index for expressing longevity in Hodgkin's disease.

The five-year survival, the standard measure of survival in neoplastic disorders, has particular value in the assessment of survival in unicentric neoplasms and may indicate in many instances that the patient who has survived for five years has a reasonable expectation of being cured of his disease. In multicentric neoplasms, however, the concept of cure rarely arises and as this is the usual concept of the aetiology of Hodgkin's disease, the five-year survival figures have lessened significance. In this series of patients 32 per cent survived for more than from the time of diagnosis. Survival statistics from the majority of studies of survival in Hodgkin's disease are expressed as the percentage surviving for five years.

When patients in a series are arranged in order of the length of time of their survival, the median survival time is that time survived by the middle patient of the series. If the median survival is less than five years, as it is in this series, it will be unaffected by those patients who survive longer than five years and will also be unaffected by those patients still alive five or more years after diagnosis. For purposes of comparison we believe it to be the most useful single index of survival. The median survival was 26·5 months. If the series is divided into two groups—1946–50 and 1952–56, the median survival was found to be 32 months for the earlier and 22 months for the latter group. These figures would seem to indicate the paucity of progress made in the treatment of Hodgkin's disease during the period under study. Survival in a group of patients with Hodgkin's disease may also be shown as a time-survival graph (Fig. 5). This distribution can be divided roughly into three parts. Approximately one-third of the patients died in the first year, one-third survived for more than one but less than five years, and approximately one-third survived five years or more.

The effect of sex on survival was studied by Epstein (1939) who observed that the disease was not only less frequent but also less malignant in women than in men. Warwick and Sellars (1959) showed that 28·6 per cent of males survived five years while 31·4 per cent of females survived for a similar period. Females survived for longer periods than males in nearly all reported series, but in most the difference was not recorded as being significant statistically. One exception however was the report by Hohl, Sarasin and Bessler (1951) who found a five-year survival of 18 per cent among 67 males and 42 per cent among sixty females.

In this study, females showed a longer survival than males ; the median survival of 49 females was 41 months whereas for the 101 males it was 24 months. Of the females 38·8 per cent survived for more than five years ; the comparable figure for males was 26·7 per cent. This percentage difference does not amount to twice the standard error of the difference of the two proportions however (it is 1·5 times as large), and cannot be regarded in itself as being of statistical significance. As many other series (Shimkin, Opperman, Bostick and Low-Beer, 1955 ; Peters, 1960 ; Epstein, 1939) record similar findings, it is probable that the difference observed between the survival of males and the survival of females in Hodgkin's disease is indeed a real one.

The effect of age on survival is always difficult to assess as the older the individual the shorter is his expectation of life, even for those individuals apparently without any disease. If a four-fold table is constructed, whereby the series is divided into those below the median age (35 years), and those of the median age

FIG. 5.—Percentage of patients surviving by yearly intervals to 5 years.

or more, and likewise, on the other axis, those who survived less than the median survival (for the purposes of calculation here approximated to 26 months), and those who survived more than this interval, the table shown in Table II results. The younger group survive longer than the older, and this to a statistically significant degree ($P < 0{\cdot}001$). This table permits one to say that a relationship exists between survival in Hodgkin's disease and the age at onset.

TABLE II.—*Survival in Certain Age Groups*

| | Survival | |
|---|---|---|
| Age | Less than 26 months | More than 26 months |
| Less than 35 | 23 | 50 |
| More than 35 | 51 | 26 |

In view of the difference in sex and survival and the possible influence of endocrine factors, an attempt was made to relate survival with the sex and age of the patient : 45 years of age arbitrarily was chosen as being the time when many women begin to undergo menopausal changes. The findings are shown in Table III and indicate again that the young survive for longer periods than the old ; this difference is more pronounced in females than in males. Females under 45 years have a better survival than their male counterparts. Females over the age

TABLE III.—*Survival in Relation to Sex and Age*

FEMALES

| Age | Survival: Less than 26 months | Survival: More than 26 months |
|---|---|---|
| Less than 45 | 7 | 24 |
| More than 45 | 14 | 4 |

MALES

| Age | Survival: Less than 26 months | Survival: More than 26 months |
|---|---|---|
| Less than 45 | 22 | 37 |
| More than 45 | 31 | 11 |

of 45 show a shorter survival than males of a similar age. Although the numbers in the various subcategories are too small to permit drawing any definite conclusions, such as they are, they further support the possibility that endocrine factors may have a bearing on the question of survival.

When survival is related to the stage of the disease, a consistent correlation can be obtained. The median survival in patients with stage 1 disease was 54 months, in stage 2, 36·5 months and in stage 3, 6 months. These figures support the accuracy of staging as a prognostic indicator. The close correlation between survival and splenic enlargement however indicates the importance of this finding as regards the accuracy of the clinical staging. For those 111 patients in whom no splenic enlargement was detected at the time of diagnosis the median survival was 36 months. The edge of the spleen was palpable a distance less than 6 cm. below the left costal margin in 22 patients who had a median survival of 12·5 months. In 4 patients with greater splenic enlargement the median survival was 3 months. Adequate clinical information was not available in 13 patients. Other studies (Hilton and Sutton, 1962) have indicated that clinical staging is helpful in prognosis in Hodgkin's disease, but not in other types of lymphoma.

### DISCUSSION

Many attempts have been made to assess the importance of various factors upon survival in Hodgkin's disease. Most of these series of patients have been derived from the summation of the experience of a large hospital or clinic or from the radiotherapy department of such institutions ; such series are not unselected and doubts can be expressed about the validity of results so derived. Selection factors in hospital or clinic experience may include restriction of admission to or preference for certain age, social or racial groups, the presence at the hospital of physicians specially interested in certain diseases, variation in the diagnostic and radiotherapy facilities and attitudes and customs among local populations and physicians. So long as it is not known what proportion of the total population is admitted to these hospitals or clinics and on what basis the admissions are arranged, statistics obtained from hospital records can be criticized on the grounds of case selection (Dameshek and Gunz, 1958). The way in which case selection occurred differed in several reports. For example, Diamond (1958) reported the experience of the Memorial Centre for cancer and allied diseases and found that more than 50 per cent of the patients in his series were not only in an advanced stage of the disease but also had received treatment in other institutions and by other physicians. He noted that the services of the Memorial Centre had been sought frequently as a last resort. Under such circumstances one would agree that these factors would have a " depleting effect on overall end results " and it is not unexpected that only 19·5 per cent of these patients survived for 5 years from the time of diagnosis. On the other hand, it is extremely difficult to ascertain what the results of such a study indicate. It is not possible to take a series such as this and make some compensatory calculation by which the results can be extrapolated to represent the findings in an unselected series of patients.

Paterson and Paterson (1954) found a crude 5-year survival of 25 per cent of 256 patients with Hodgkin's disease treated between 1934 and 1947 at the Christie Hospital in Manchester ; they reported an overall crude 5-year survival of 25 per cent. As this series was derived from the experience of a radiotherapy institute it is probable that the series included a high proportion of patients referred for this form of management and so should be regarded as being selected. Many other series of patients with Hodgkin's disease have been reported from radiotherapy centres (Hilton and Sutton, 1962 ; Elkins, 1956 ; Peters, 1950).

The results reported by Peters (1960) may also be criticized from the standpoint of case selection. The survival of 285 patients with Hodgkin's disease treated by irradiation in Toronto between 1924 and 1954 were reported. Twenty patients were treated in the period 1924 and 1934, and 60 per cent of these survived for 5 years. In the 5-year period between 1950 and 1954, however, 88 patients were treated with a 5-year survival of 29 per cent. It is apparent that the number of patients being treated has increased by almost 9 times while the survival shows a decrease. It would seem logical to assume that with the larger number of patients a more representative sampling of the disease is being obtained. The remarkable survival in Peters' early series may be explained by case selection.

Cook, Krabbenhoft and Leucutia (1959) reported the results of treatment of 392 patients with Hodgkin's disease but excluded 45 patients who had received no treatment. They analyzed the results of treatment in these 347 treated patients and found that 34·6 per cent survived for more than 5 years from the time of diagnosis. The fact that they excluded patients from the analysis of survival is again evidence of case selection. One might with justification presume that it these exclusions had not been made the overall survival would not have been as long as that reported. Criticism on the grounds of case selection can be made of many other reported series.

The interpretation of the histopathologist may greatly affect the survival of a series of patients diagnosed as having Hodgkin's disease and constitutes yet another means by which selection of cases may occur. In those cases where difficulty is experienced in differentiating between hyperplastic conditions and Hodgkin's disease if any bias towards a diagnosis of Hodgkin's disease occurs, such a series subsequently will show extended survival. Review of the histopathology of all patients with Hodgkin's disease who live for more than 5 years and especially if they have shown no further evidence of disease, is highly desirable. Differences in classification of the malignant lymphoid tumours may vary the composition of series of patients diagnosed as Hodgkin's disease. Comparability between series may thus be lost.

For these reasons it is difficult or even impossible to make a valid comparative analysis of the results of treatment in Hodgkin's disease at different centres. Even if one ignores factors of selection one finds that survival is measured by some from the date of diagnosis (Videbaek, 1950) and by others from the time of onset of symptoms (Hohl, Sarasin and Bessler, 1951). It would seem advisable that in future studies survival be measured from the time of diagnosis. This is at least a definite date. Often the onset of symptoms of Hodgkin's disease is insidious and patients may have difficulty in estimating their duration accurately. Many patients " round off " the duration of the symptoms into such periods as one, three, six or nine months or one year, but rarely do they estimate the intervening periods. It is less accurate to measure survival from the time of onset of symptoms than from the time of diagnosis. As each method of expressing survival measures different features, it would also seem to be advisable to express survival as the mode, the median, the mean (taking cognizance of the problem of those patients still alive), the percentage surviving for 5 years and the time survival graph methods for each series of patients.

Other absolute 5-year survival figures reported recently include 19·3 per cent (Bethell, Andrews, Neligh and Meyers, 1951), 32·1 per cent (Hohl, Sarasin, Bessler, 1951), 32·6 per cent (Kaplan and Allen, 1952), 25 per cent (Nice and Stenstrom, 1954 ; Elkins, 1956), 18·6 per cent (Shimkin, Oppermann, Bostick and Low-Beer, 1955), and 31 per cent (Hilton and Sutton, 1962). In this series 32 per cent survived for 5 years but as already indicated comparative analysis is of dubious validity.

The effect of any treatment on the survival of patients with Hodgkin's disease is extremely difficult to assess. In this series the general principles governing treatment of patients with Hodgkin's disease have remained unchanged during the period under study. Radical radiation therapy has been given to those patients with stage 1 disease and to some patients with stage 2 disease where the glandular involvement was limited to adjacent areas. Patients with widespread stage 2 disease and all patients with stage 3 disease had treatment with chemotherapy, radiotherapy or both. The majority of patients in stage 3 had nitrogen mustard as primary treatment. Adrenal steroids were used especially in patients with anaemia. Many patients, especially in the terminal stages of their illness, received a multiplicity of therapeutic agents, sometimes singly, and sometimes in combination. If any response to treatment was obtained under these circumstances, it was possible only to guess which agent had been responsible and it was not possible to exclude the occurrence of a natural remission to explain the improvement.

While the clinical course of Hodgkin's disease is usually progressive with a fatal termination, there may be very considerable variation in the rate of progress when one patient is compared with another. There may be similar variation however in the same patient at different times. Natural remissions and variation in the rapidity of progress of the disease occur and often are unassociated with treatment. Claims that therapy has been responsible for improvement, therefore, have to be made with caution. Any attempt to analyze the effect of a particular form of treatment on the duration of survival in Hodgkin's disease must take cognizance of the fact that two factors are operative—the biological activity of the disease and the effect of treatment. Frequently these factors cannot be separated although in those patients with Hodgkin's paragranuloma the histological appearances may give an indication of the improved prognosis by virtue of the less active form of the disease.

From this series of patients it is difficult to draw firm conclusions. The results of Shimkin *et al.* (1955) and of Hohl *et al.* (1951) however, show significant differences between the duration of the illness in females and males, females surviving for longer periods of time. While our results show this difference it is not significant statistically. Our results suggest that while this difference may be present in younger females and males the pattern of difference is reversed after the age of 45 and that older females survive for shorter periods than older males. As Shimkin has suggested, it would be interesting to carry out a clinical study on the effect of hormone therapy in an attempt to prolong survival. Oestrogen therapy for the post-menopausal female might be the most obvious first choice. When the patient reaches the terminal stages of Hodgkin's disease we do not possess such a wealth of effective therapeutic agents that would allow any factor of possible benefit to be ignored. The assessment of the results of such a clinical trial, however, would be extremely difficult.

It is to be hoped that new and better agents for the treatment of Hodgkin's disease will be developed in the years to come. This hope, together with the albeit relative successes obtained with present day management must support the energetic employment of all means of treatment at our disposal in this disease.

With regard to philosophic considerations we would not presume to try to improve upon the statement of Diamond (1958) who concluded his report: " In no sphere of cancer medicine is there such an appallingly nihilistic philosophy among physicians and surgeons, and even among radiologists, as there is in the treatment of patients with malignant lymphomas. Yet we see swift and even enthusiastic treatment by radical surgery of cancers of the lung, pancreas, stomach, soft parts, and bone, to name a few, whereas the total experience in terms of five-year survival rates for *all* such patients hovers somewhat below 10 per cent!

Let us be critical of ourselves, of our present methods of treatment and of our instructions to our colleagues and students and impart a *dynamic* philosophy in treatment. Let us continue to search out the past experiences, the natural history, and the vagaries of the biological activity of Hodgkin's disease while we press forward in our search for better radiotherapeutic and chemotherapeutic measures for this disease.

In the meantime, let us continue to prevail on our medical colleagues to attempt diagnosis of Hodgkin's disease early, without temporization and guesswork, so that the therapeutic means now available to us can be used forthwith and to the best advantage of the patient ".

### SUMMARY

One hundred and fifty residents of the Province of Saskatchewan were found to have had Hodgkin's disease between 1946 and 1956 (inclusive). As all known cases of Hodgkin's disease were collected from a clearly defined geographic area during a specified period of time, it is suggested that this series is unselected. The methods by which the series was assembled are described together with the unusual opportunities which exist in Saskatchewan for such a study.

The mean survival for patients in this series was 26·5 months and the mean survival 30·6 months. Thirty-two per cent survived 5 years from the time of diagnosis. The mode of survival was 0–3 months. Approximately one-third of the patients died within one year after diagnosis, one-third survived for more than one but less than five years, and one-third survived for five years or more.

As there is no universally accepted classification of malignant lymphoid tumours it is probably that Hodgkin's disease is an ill-defined histopathological entity. Selection of cases has occurred in a variety of ways in many of the previously reported series of patients with Hodgkin's disease. For these reasons it is suggested that comparative analysis between one reported series and another is probably not valid.

The results of this series indicate that females survive for longer periods than males. Survival was longer in the younger age groups. Endocrine factors may be important in the survival of patients with Hodgkin's disease and for the elderly female patient a clinical trial with oestrogens would appear to be justifiable.

### REFERENCES

BETHELL, F. H., ANDREWS, G. A., NELIGH, R. B. AND MEYERS, M. C.—(1951) *Amer. J. Roentgenol.*, **64**, 61.
COOK, J. C., KRABBENHOFT, K. L. AND LEUCUTIA, T.—(1959) *Ibid.*, **82**, 651.
DAMESHEK, W. AND GUNZ, F.—(1958) 'Leukemia'. New York (Grune and Stratton Co.), p. 30.
DIAMOND, H. D.—(1958) *Ann. N.Y. Acad. Sci.*, **73**, 357.
ELKINS, H. B.—(1956) *Amer. J. Roentgenol.*, **76**, 960.
EPSTEIN, E.—(1939) *Amer. J. Cancer*, **35**, 230.
FOX, H—(1926) *Ann. med. Hist.*, **8**, 370.
GARRISON, F. H.—(1939) 'History of Medicine'. Philadelphia (W. B. Saunders Co.), p. 423.
GILLIAM, A. G.—(1953) *Blood*, **8**, 693.
GREEN, I., INKELAS, M. AND ALLEN, L. B.—(1960) *Lancet*, i, 30.
HARRISON, C. V.—(1952) *J. Path. Bact.*, **64**, 513.
HILTON, G. AND SUTTON, P. M.—(1962) *Lancet*, i, 283.
HOHL, K., SARASIN, P. AND BESSLER, W.—(1951) *Oncologia*, **4**, 1.
HOSTER, H. A.—(1944) *Ohio J. Sci.*, **44**, 245.
*Idem*, DRATMAN, M. B., CRAVER, L. F. AND ROLNICK, H. A.—(1948) *Cancer Res.*, **8**, 1.
JACKSON, H., Jr.—(1937) *Surg. Gynec. Obstet.*, **64**, 465.
*Idem* AND PARKER, F., Jr.—(1944) *New Engl. J. Med.*, **230**, 1.
KAPLAN, I. I. AND ALLEN, L. L.—(1952) *Int. Rec. Med.*, **165**, 11.
MACMAHON, B.—(1957) *Cancer*, **10**, 1045.
MAJOR, R. H.—(1932) 'Classic Descriptions of Disease'. Springfield (Charles C. Thomas Co.), p. 183.
MEIGHAN, S. S.—(1961) *Canad. med. Ass. J.*, **84**, 631.—(1963) *Cancer* (in press.)
NICE, C. M. AND STENSTROM, K. W.—(1954) *Amer. J. Roentgenol.*, **62**, 641.
PATERSON, R. AND PATERSON, E.—(1954) *Brit. med. J.*, ii, 1315.
PETERS, M. V.—(1950) *Amer. J. Roentgenol.*, **63**, 299.—(1960) Fourth National Cancer Conference Proceedings, Philadelphia (J. B. Lippincott Co.), p. 571.
SHIMKIN, M. B., OPPERMANN, K. C., BOSTICK, W. L. AND LOW-BEER, B. V. A.—(1955) *Ann. intern. Med.*, **42**, 136.
VIDEBAEK, A.—(1950) *Acta med. scand.*, **136**, 203.
WARWICK, O. H. AND SELLARS, A. H.—(1959) *Canad. med. Ass. J.*, **80**, 423.
WATSON, T. A.—(1950) *Canad. J. Publ. Hlth*, **41**, 308.
WILLIS, R. A.—(1960) 'Pathology of Tumours', 3rd edition. London (Butterworth Co.), p. 764.—(1961) 'The Principles of Pathology', including Bacteriology, 2nd edition. London (Butterworth Co.), p. 549.